# Rational Design of Polyamine-Based Cryogels for Metal Ion Sorption

**DOI:** 10.3390/molecules25204801

**Published:** 2020-10-19

**Authors:** Irina Malakhova, Yuliya Privar, Yuliya Parotkina, Aleksandr Mironenko, Marina Eliseikina, Denis Balatskiy, Alexey Golikov, Svetlana Bratskaya

**Affiliations:** 1 Institute of Chemistry, Far Eastern Branch of Russian Academy of Sciences, 159, prosp.100-letiya Vladivostoka, 690022 Vladivostok, Russia; newira94@gmail.com (I.M.); privar.juliya@gmail.com (Y.P.); azarova.87@mail.ru (Y.P.); almironenko@gmail.com (A.M.); denis.balatskiy@bk.ru (D.B.); glk@ich.dvo.ru (A.G.); 2 A.V. Zhirmunsky National Scientific Center of Marine Biology, Far Eastern Branch of Russian Academy of Sciences, 17, Palchevskogo street, 690041 Vladivostok, Russia; meliseikina@yandex.ru

**Keywords:** polyallylamine, polyethyleneimine, cryogel, sorption kinetics, sorption dynamics, metal ions, ferrocyanides, composite sorbents

## Abstract

Here we report the method of fabrication of supermacroporous monolith sorbents (cryogels) via covalent cross-linking of polyallylamine (PAA) with diglycidyl ether of 1,4-butandiol. Using comparative analysis of the permeability and sorption performance of the obtained PAA cryogels and earlier developed polyethyleneimine (PEI) cryogels, we have demonstrated the advantages and disadvantages of these polymers as sorbents of heavy metal ions (Cu(II), Zn(II), Cd(II), and Ni(II)) in fixed-bed applications and as supermacroporous matrices for the fabrication of composite cryogels containing copper ferrocyanide (CuFCN) for cesium ion sorption. Applying the rate constant distribution (RCD) model to the kinetic curves of Cu(II) ion sorption on PAA and PEI cryogels, we have elucidated the difference in sorption/desorption rates and affinity constants of these materials and showed that physical sorption contributed to the Cu(II) uptake by PAA, but not to that by PEI cryogels. It was shown that PAA cryogels had significantly higher selectivity for Cu(II) sorption in the presence of Zn(II) and Cd(II) ions in comparison with that of PEI cryogels, while irreversible sorption of Co(II) ions by PEI can be used for the separation of Ni(II) and Co(II) ions. Using IR and Mössbauer spectroscopy, we have demonstrated that strong complexation of Cu(II) ions with PEI significantly affects the in situ formation of Cu(II) ferrocyanide nanosorbents leading to their inefficiency for Cs^+^ ions selective uptake, whereas PAA cryogel was applicable for the fabrication of efficient monolith composites via the in situ formation of CuFCN or loading of ex situ formed CuFCN colloids.

## 1. Introduction

Polyamines are widely used in environmental applications as flocculants, complexing agents for ultrafiltration, and different types of sorbents and ion-exchangers. Polyethyleneimine (PEI) and polyallylamine (PAA) are among most widely used and affordable polyamines, which have been applied for the removal of dyes [1], metal ions [2,3,4,5,6,7,8,9,10], and other pollutants from natural and wastewaters, as well as for CO_2_ uptake from the air [11,12,13]. Although biobased polymers, such as chitosan, have advantages in terms of biodegradability, PEI is one of the most efficient chelating polymers commercially available for large-scale water treatment technologies. In ligand-assisted separation of heavy metal ions using membrane ultrafiltration, PEI showed higher efficiency than chitosan for the recovery of Hg(II) [4], Cu(II), and Ni(II) [5].

Due to the presence of reactive primary amino groups, insoluble sorbents can be obtained from PEI and PAA via covalent cross-linking with different type of reagents, including epichlorohydrin [14], diglycidyl ethers of glycols [9,15,16,17], and glutaraldehyde [16]. The application of functional cross-linkers [18] or N- and S-containing reagents allowed the fabrication of PAA- and PEI-based materials with enhanced sorption properties toward different metal ions [14,19,20,21]. PAA hydrogels with thiourea groups have been reported for efficient Hg(II) removal in a batch [18]; PAA beads modified with glucose were suggested for boron adsorption [17]. Insoluble materials can be also obtained from PAA and PEI via self-assembly with oppositely charged polymers and colloids, e.g., a layer-by-layer (LBL) composite of PAA and graphene was fabricated for the detection of heavy metal ions [22]. Although PAA is a less efficient chelator for heavy metal cations than PEI is, it is characterized by the high density of primary amino groups per weigh unit and showed high efficiency of dyes and toxic and precious metals in anionic forms [10,23]. Due to the high reactivity of amino groups, both PAA and PEI can be easily functionalized, e.g., PAA and PEI were carboxymethylated to fabricate chelating fibers [24] modified with 2-vinylpyridine [14] and 5- methyl-4-imidazolylmethanol [19] to enhance their binding capacity for heavy and noble metal ions. We have demonstrated that, depending on the type of functionalization reaction, PAA or PEI can serve as a more suitable matrix for the fabrication of the functional materials. In the Michael reaction with 2-vinylpyridine, PEI was less reactive than PAA due to the branched structure and the presence of not only primary but also secondary and tertiary amino groups [14]. However, the activity of PAA in side cross-linking reactions, which accompanied hydroxyl group nucleophilic substitution in 5-methyl-4-imidazolylmethanol “in gel”, made it a less suitable polymer precursor than the branched PEI [19]. At the same time, imidazolylmethylation of pre-formed PEI cryogel resulted in a loss of its mechanical stability [19]. Therefore, the selection of the appropriate polymer matrix for post-functionalization is not always a straightforward task.

One of the crucial points in the application of sorbents is its performance in a column. While weakly cross-linked hydrogels can have high sorption capacities, they can hardly be applicable in real systems due to the lack of mechanical stability. Recently, supermacroporous monolith materials (polymeric cryogels) have attracted attention as promising materials for water treatment and disinfection [25,26,27,28,29,30]. Due to the interconnected 3D porous structure with a wall thickness of only a few microns, these materials show the ultimate kinetic characteristics in fixed-bed application [31]. Furthermore, the large pore size of cryogels (up to hundreds of microns) allows the fabrication of organic–inorganic cryogels for the selective removal of highly toxic metal and metalloid ions without blocking solution flow in monoliths [32,33]. Immobilization of inorganic sorbent nanoparticles in the polymer matrix is one of the most efficient approaches to the recovery of most toxic inorganic pollutants such as mercury [34,35] and arsenic [32] species. Since polyamines do not constitute a chemically inert matrix and can chemically adsorb metal ions, which can serve as precursors for inorganic sorbents (transition metal oxides, hydroxides, sulfides, ferrocyanides, etc.), the composites can be formed in situ, which simplifies the fabrication method and allows better control of the composite morphology. However, it is important to take into account that the stability of the complexes of precursor metal ions with a polymeric matrix can significantly affect the composition and properties of nanoparticles [36,37].

Here we report on the fabrication of PAA cryogels and their sorption properties toward metal ions in a batch and a fixed-bed in comparison with earlier developed PEI cryogels [15]. Aside from the focus on the difference in fabrication conditions and sorption selectivity to rationalize the choice of the polymer for certain applications of cryogels, we investigate how chelating properties of these N-donor ligands affected the formation and performance of supermacroporous composites containing copper ferrocyanide for cesium ion sorption.

## 2. Results and Discussion

### 2.1. Fabrication and Characterization of the Monolith PAA Cryogels

**Figure 1:**
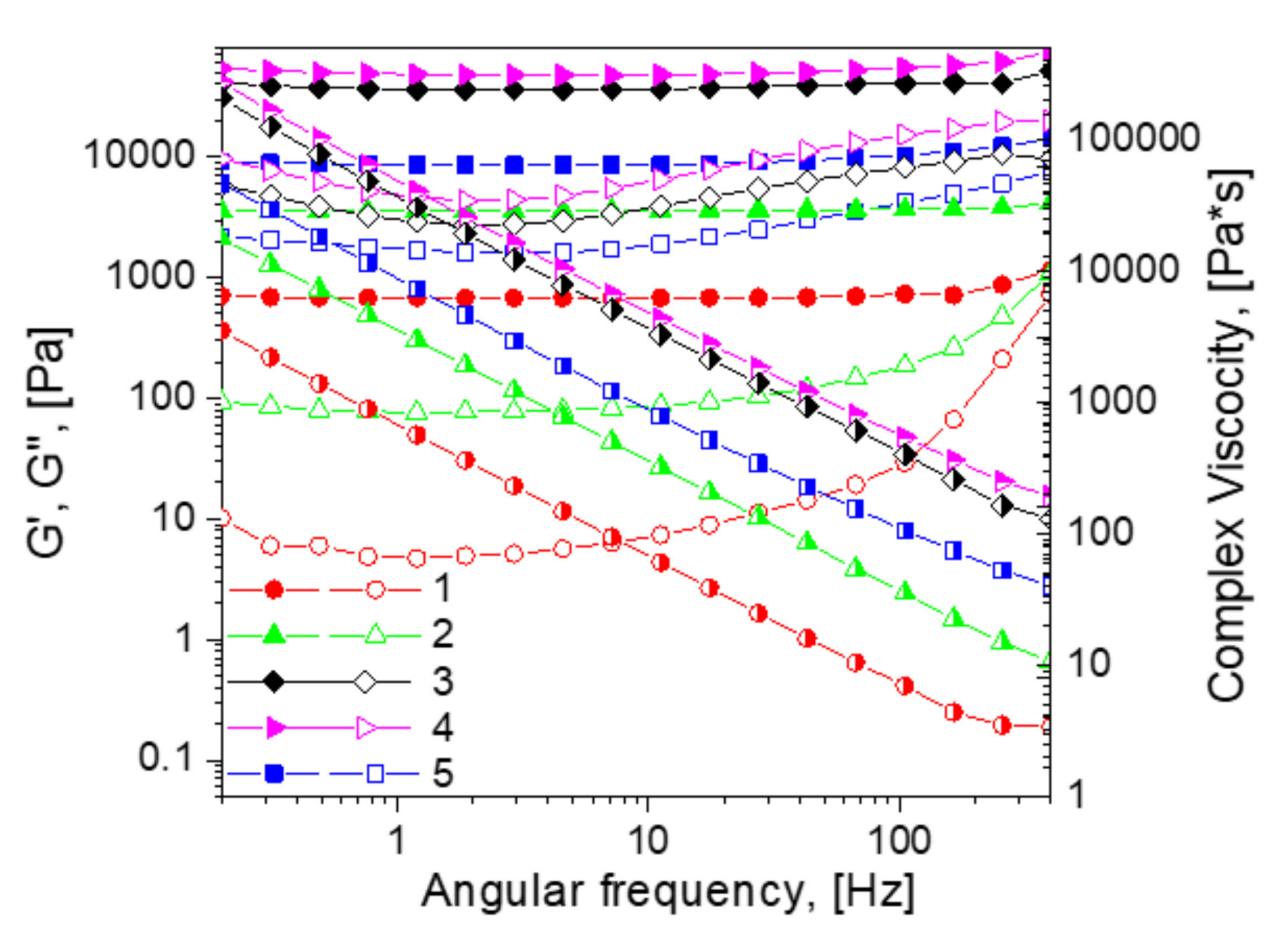
Mechanical spectra of polyallylamine (PAA) and polyethyleneimine (PEI) gels and cryogels obtained at different cross-linker (DGEBD):polymer ratios using 5% polymer solutions: 1—DGEBD:PAA 1:8, T = 25 °C, 24 h; 2—DGEBD:PAA 1:8, T = −20 °C, 24 h; 3—DGEBD:PAA 1:8, T = −20 °C, 72 h; 4—DGEBD:PAA 1:8, T = −20 °C, 168 h; 5—DGEBD:PEI 1:4, T = −20 °C, 168 h. Filled symbols represent storage modulus (G′), open symbols represent loss modulus (G′′), half-filled symbols represent complex viscosity.

Using our experience in the fabrication of PEI cryogels covalently cross-linked with different diglycidyl ethers of glycols [15], we have selected diglycidyl ether of 1,4-butandiol (DGEBD) as a cross-linker for PAA cryogels, since this commercially available and affordable reagent yielded PEI cryogels with optimal sorption and mechanical properties. Unlike PEI with primary, secondary, and tertiary amino groups in its structure, PAA contains only primary amino groups, whose deprotonation results in a loss of PAA solubility at pH > 7.4. As we have shown earlier for PEI [16] and chitosan [38], the rate of cross-linking reaction between polyamines and diglycidyl ethers of glycols decreases with the decrease of pH. However, in cryogel fabrication, since one must avoid phase separation before solvent crystallization and polymer covalent cross-linking, PAA cryogels were fabricated at pH 7.4. The mechanical spectra of PAA recorded within 24 h after addition of the cross-linker (DGEBD) have confirmed the formation of strong gels with ratios of storage (G′)/loss(G′′) modulus >10 (Figure 1). Cross-linking for 24 h at subzero temperature yielded a stronger PAA gel compared to that obtained at room temperature, which can be related to the cryoconcentration effect [39]. Evolution of the mechanical spectra of PAA cryogels observed over 7 days showed that the most significant changes in rheological properties of the gel occurred in the period between 24 and 72 h after cross-linking addition. However, to ensure the completeness of the cross-linking reaction, cryogels cross-linked for 7 days were used in all experiments. 

**Scheme 1:**
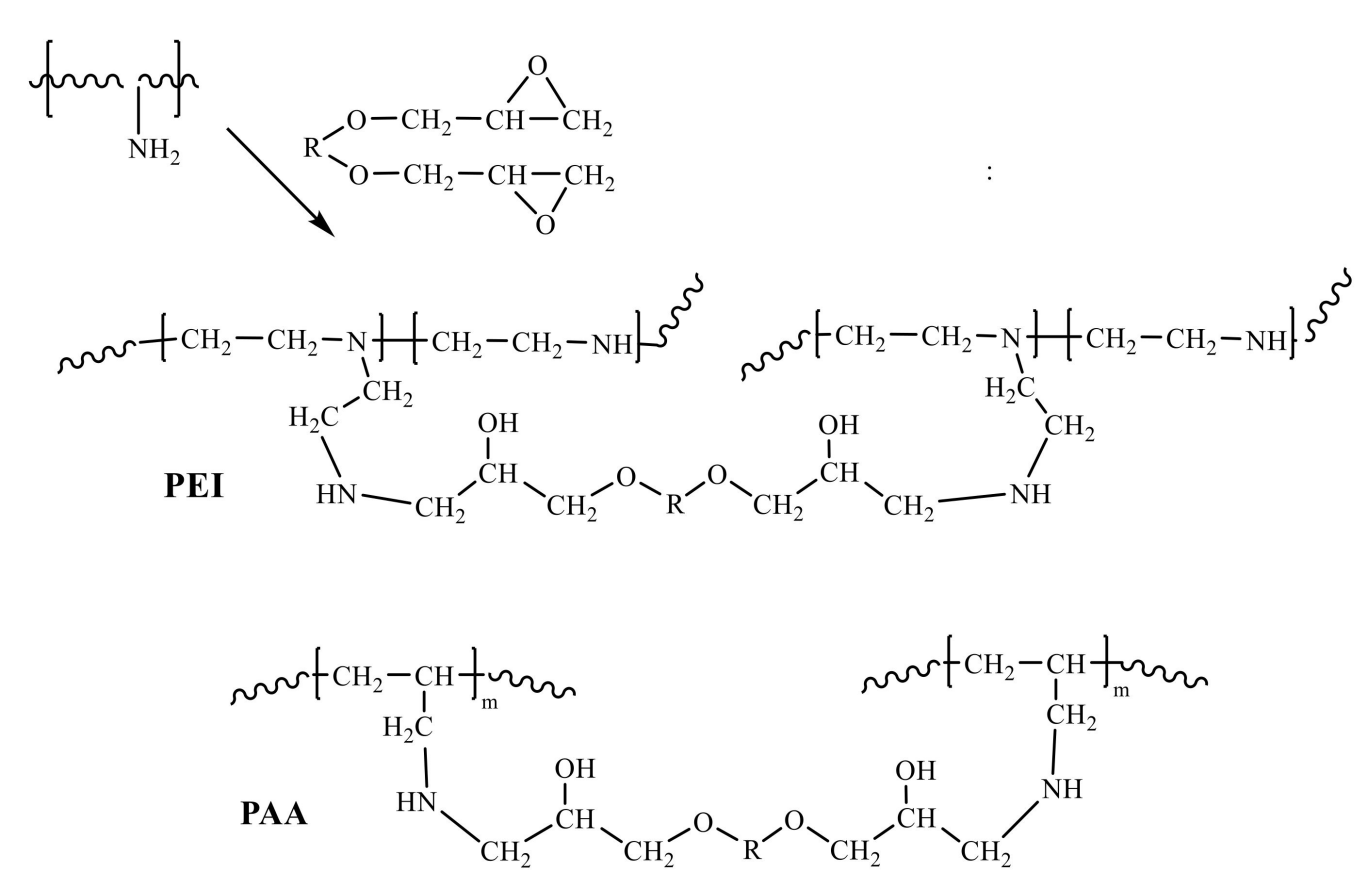
Scheme of polyethyleneimine (PEI) and polyallylamine (PAA) cross-linking with diglycidyl ethers of glycols.

Since the irregular structure of the branched PEI does not allow unambiguous determination of the molecular weight of the monomer unit with one primary amino group, we have compared mechanical properties of PAA and PEI cryogels cross-linked at DGEBD:polymer molar ratios of 1:8 and 1:4, respectively, so that the weight ratios of cross-linker and polymer would be comparable Table S1 (Supplementary Materials). The cross-linking scheme that was assumed for molar ratios calculations is shown in Scheme 1.

**Figure 2:**
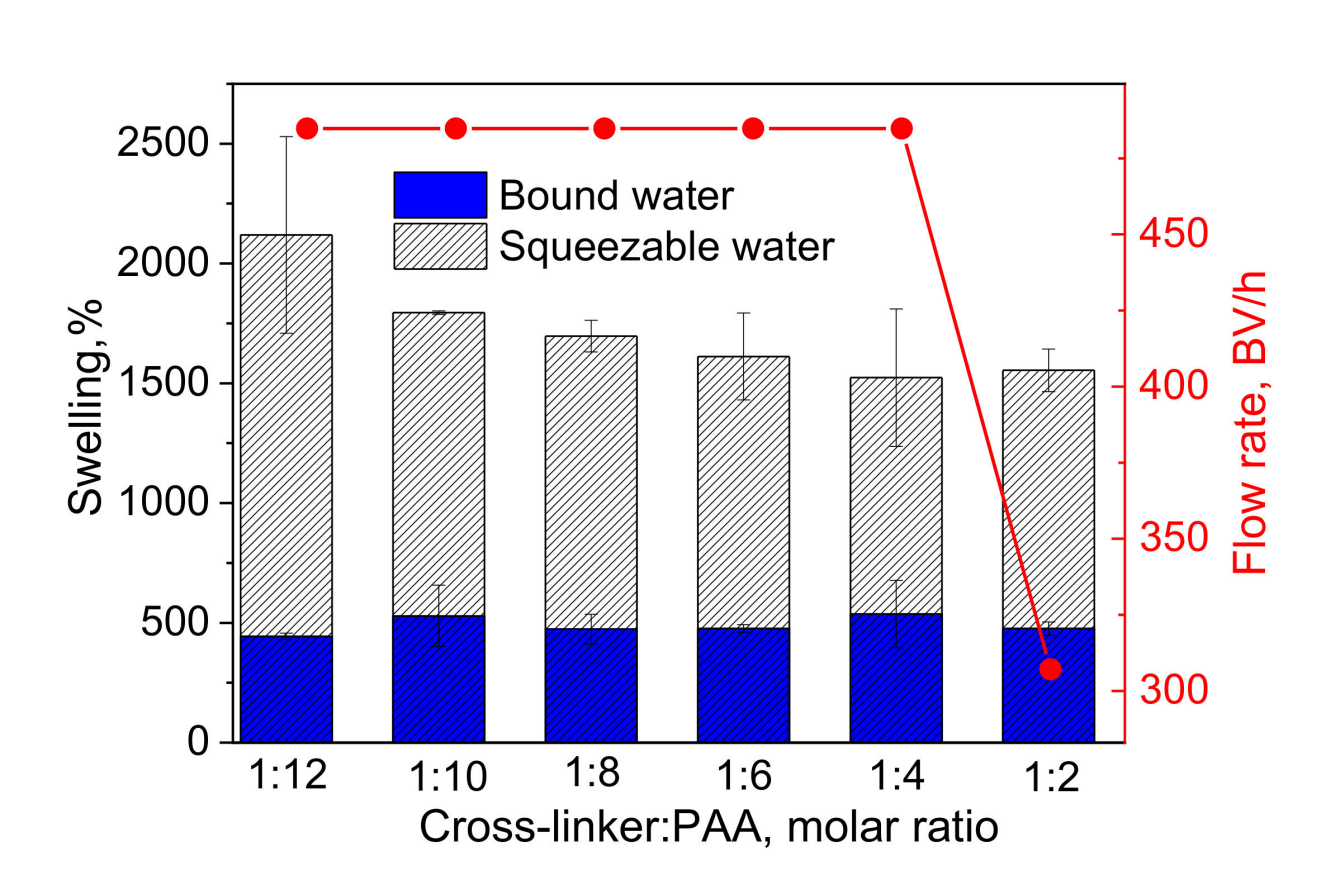
Swelling and permeability of PAA cryogels cross-linked with DGEBD.

To achieve an optimum balance between the sorption performance and mechanical properties of PAA cryogels, the cross-linker:polymer molar ratio was varied from 1:2 to 1:12. Figure 2 shows that squeezable (freely flowing) water makes the main contribution to the swelling of PAA cryogels at all cross-linking degrees, which determines very good permeability of the materials, supporting a flow rate of at least 500 bed volumes (BV)/h (the flow rate upper limit of the pump used) at DGEBD:PAA molar ratios from 1:4 to 1:12. The PAA cryogel permeability decreased to ~300 BV/h at the highest cross-linking degree (1:2) only and was, in general, higher than the permeability of PEI cryogels, which dropped to 9 BV/h at molar ratio DGEBD:PEI 1:6 [15] (Table S1, Supplementary Materials).

**Figure 3:**
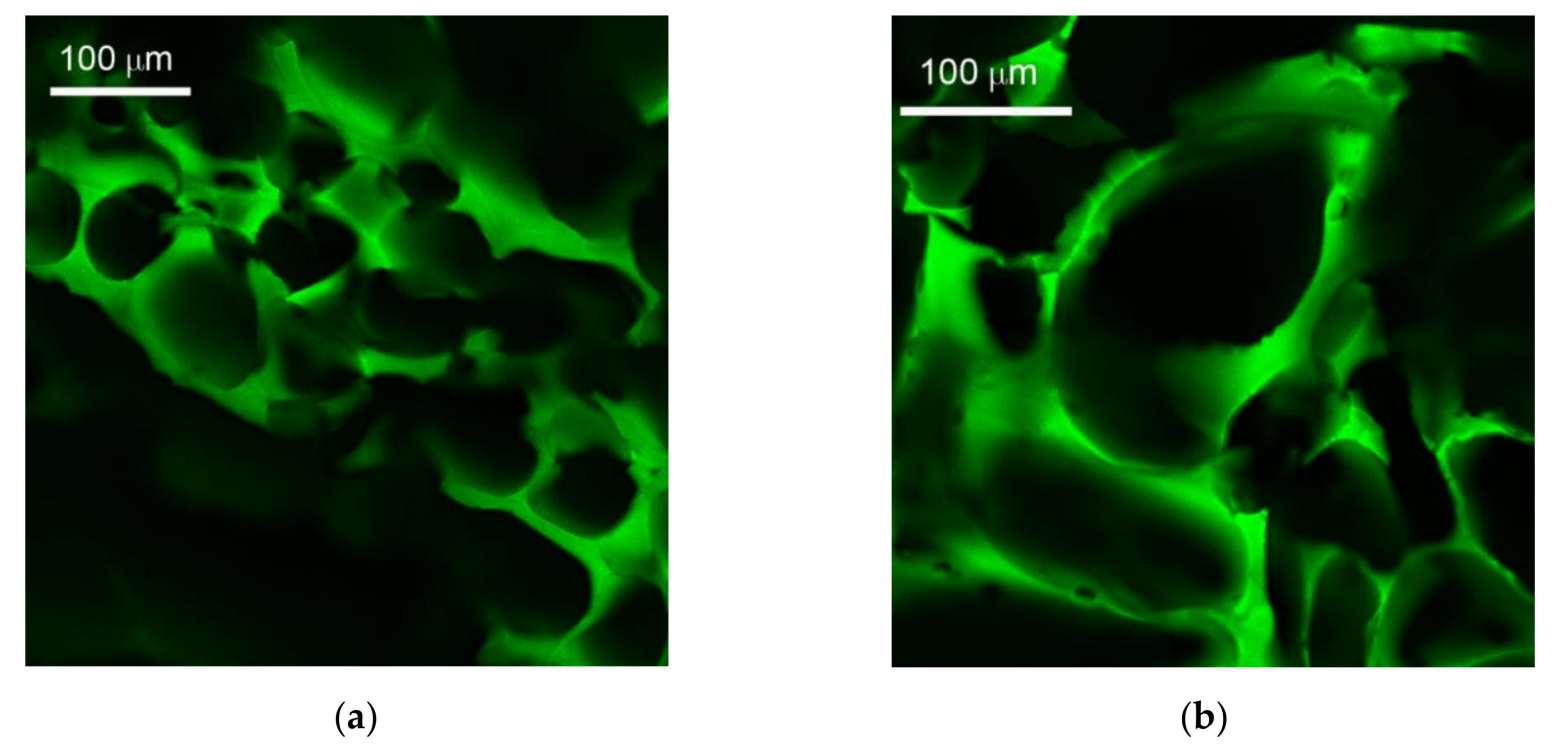
Confocal laser scanning microscopy (CLSM) images of the stained with fluorescein PAA cryogels cross-linked at DGEBD:PAA molar ratios of 1:2 (**a**) and 1:12 (**b**).

Confocal microscopy confirmed a supermacroporous structure of the PAA cryogels (Figure 3). The average pore sizes for the swollen PAA cryogels in the free base form were 113 ± 29 and 71 ± 13 µm for DGEBD:PAA molar ratios of 1:12 and 1:2, respectively. The pore wall thicknesses for these cryogels were 8.7 ± 2.8 and 10.5 ± 4.5 µm, respectively. Lower pore diameters and thicker walls for PAA cryogel with the highest cross-linking degree were in good agreement with the lowest permeability of this material.

### 2.2. Sorption Properties of PEI and PAA Cryogels

**Figure 4:**
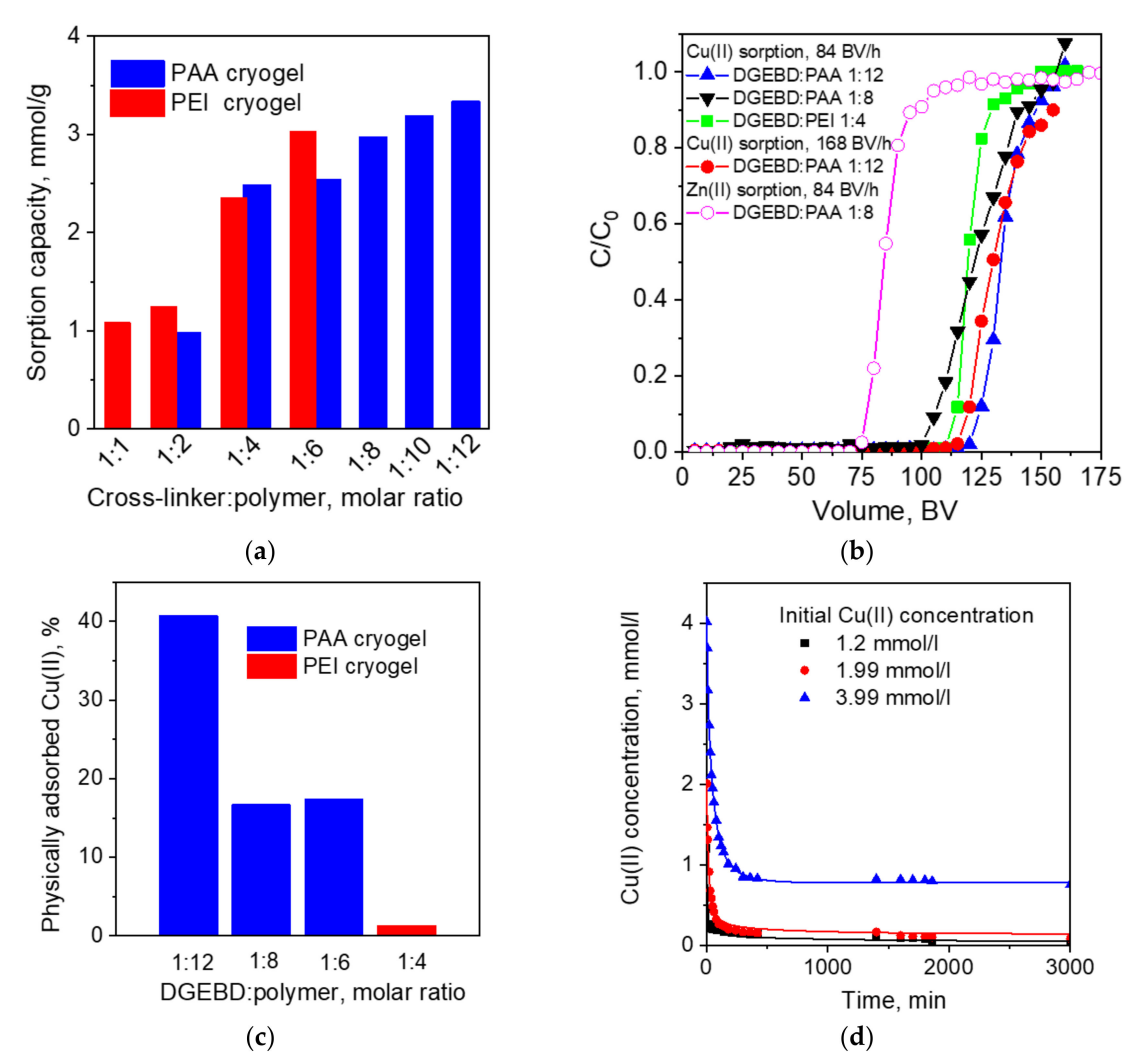
Sorption capacity for Cu(II) ions of PEI and PAA cryogels cross-linked with DGEBD, pH = 5, T = 25 °C (**a**). Breakthrough curves of Cu(II) ion sorption on PEI and PAA monolith cryogels, initial Cu(II) concentration (C_0_) was 100 mg/L, while that of Zn(II) was 75 mg/L, pH = 5, flow rate was 84 BV/h or 168 BV/h, column diameter was 0.48 cm, bed length was 6 cm (**b**). Contribution of physical sorption to the overall static sorption capacity of PAA and PEI cryogels toward Cu(II) ions (**c**). Kinetic curves of Cu(II) ions sorption on fines of PAA cryogel cross-linked with DGEBD at a molar ratio of DGEEB:PAA of 1:10, pH = 5, T = 25 °C, solid:liquid ratio was 1:1000 (**d**).

As we have shown recently [15], the supermacroporous structure of the PEI cryogels assured high accessibility of the sorption sites for Cu(II) ions under dynamic conditions, so even at high flow rates, e.g., ~170 BV/h, the overall dynamic sorption capacity was very close to the maximal static one. To elucidate an optimal cross-linking degree for the application of PAA cryogels, we have screened their sorption properties in a batch (the monolith cryogels were preliminary converted to fines to eliminate diffusion limitation) and compared the maximal sorption capacities for PAA and PEI cryogels. Cross-linking conditions significantly affected the sorption properties of the materials, e.g., the sorption capacities of PAA and PEI beads cross-linked with epichlorohydrin were 1.1 and 3.58 mmol/g, respectively [14]. Due to the high efficiency of PAA cross-linking at subzero temperature, robust PAA cryogels can be obtained at the lowest DGEBD:PAA molar ratio of 1:12, yielding materials with sorption capacities up to 3.4 mmol/g, which was higher than the maximal capacity of mechanically stable PEI cryogels (Figure 4a). Weakly cross-linked PAA cryogels (PAA-DGEBD_1:12) have also demonstrated good performance in fixed-bed (Figure 4b) allowing a Cu(II) removal efficiency of 98% from a solution containing 100 mg(Cu)/L, residual Cu(II) concentration was below 1 mg/L, as required by the World Health Organization (WHO) for drinking water. PAA cryogel was able to remove Cu(II) with the same efficiency also at flow rate of 168 BV/h, which made it comparable with PEI cryogel reported earlier [15]. However, when PEI cryogel was used, much deeper decontamination of the solution to the residual copper concentration of 0.03 mg/L was achieved (Figure 4b). PAA cryogel also showed high efficiency for the removal of Zn(II) ions (Figure 4b).

It is important to take into account that physical sorption can contribute notably to the sorption mechanism on highly swellable sorbents [40]. Figure 4c shows a significant difference in the contributions of physical sorption to overall sorption capacity for PAA and PEI cryogels. While PEI cryogels retain Cu(II) ions predominantly via chemisorption, physical sorption of Cu(II) by PAA cryogel was important for the weakly cross-linked cryogel. Since the swelling of PAA (Figure 2) and PEI cryogels [15] was comparable, the difference in sorption properties, most likely, originated from the different affinities of PAA and PEI toward Cu(II) ions. 

Recently, we have developed the extended rate constant distribution model (RCD-model) for sorption in heterogeneous systems and applied it to investigations of metal ion sorption on PEI cryogels [41]. The RCD-function, which provides complete information about sorption properties for the material with continuous distribution of the sorption sites, which can be calculated from sorption kinetic curves recorded at different solid:liquid ratios or initial concentrations of the adsorbate. Using the RCD-function, one can calculate several other distribution functions, including 2D distribution of sorption sites over constants of sorption rate (K_s_ – ρ(K_s_)); 2D distribution of sorption sites over constants of desorption rate (K_d_ − ρ(K_d_)); and 2D affinity—distribution of sorption sites over affinity constants (K_AF_ =K_s_/K_d_*Q_max_ – ρ(K_AF_)). To account for diffusion, the RCD model was modified and an additional parameter—“characteristic diffusion time” was introduced [42].

**Figure 5:**
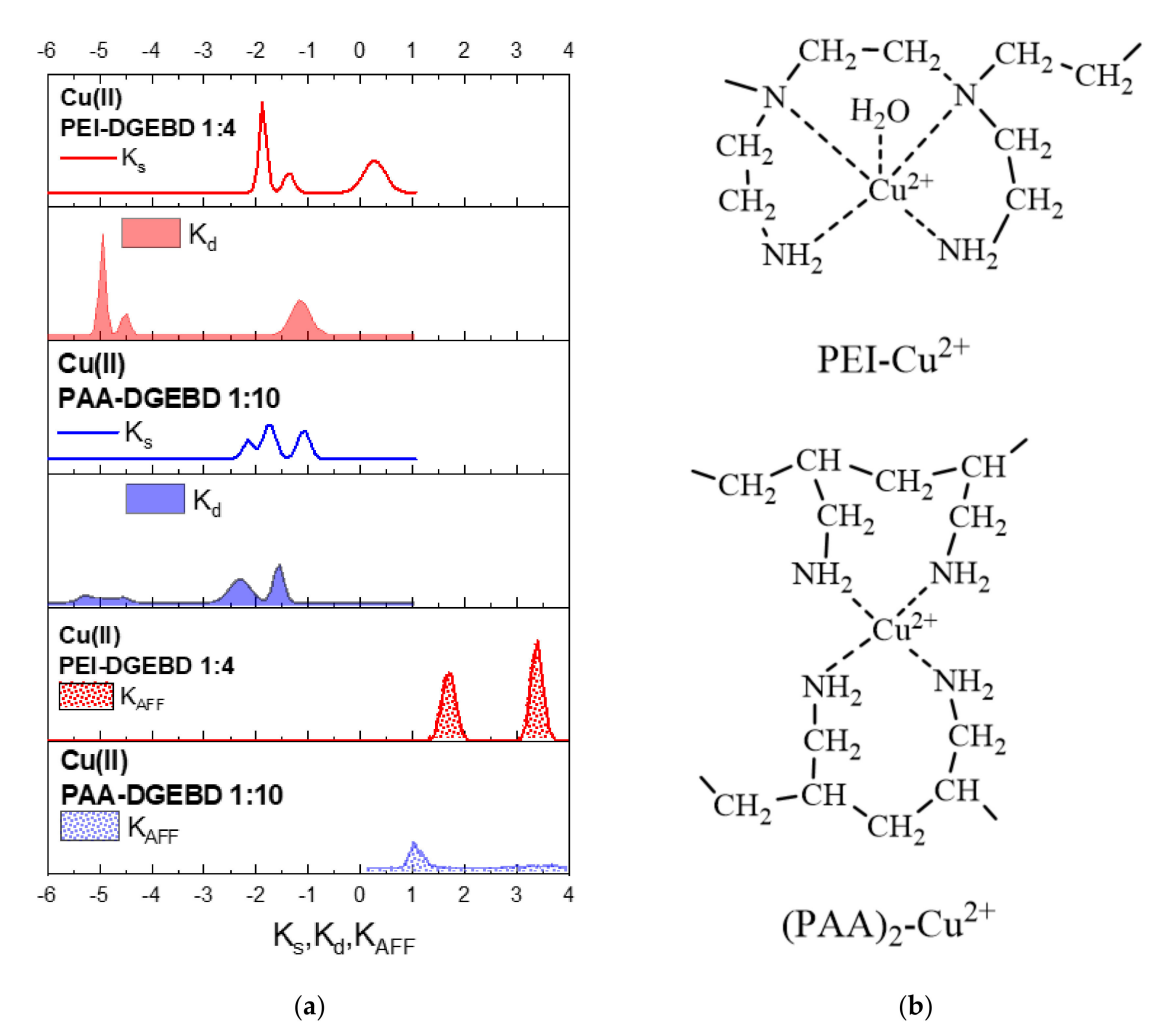
2D distributions of the Cu(II) sorption sites of PAA and PEI cryogel over the constants of sorption (K_s_) and desorption (K_d_) rates and affinity constants (K_AFF_) calculated using the rate constant distribution (RCD) model (**a**) and hypothetical structure of Cu(II) complexes with PAA and PEI (**b**).

Figure 4d shows kinetic curves of Cu(II) ion sorption on PAA cryogel cross-linked at the DGEBD:PAA mole ratio of 1:10, which were used to calculate the distribution functions for PAA cryogel (Figure 5a) applying the RCD-diffusion model [42]. Kinetic curves for Cu(II) ions sorption on PEI cryogel are shown in Figure S1, Supplementary Materials. While distribution functions for Cu(II) sorption/desorption rate and affinity constants clearly show the presence of “fast” and “slow” centers with low and high affinity for PEI cryogel, one group of the sorption centers with a broad distribution of the sorption/desorption rate constants was found for PAA. Despite the presence of only the primary amino group in the PAA structure, cross-linking affects chain flexibility and spatial distribution of the functional groups, which results in heterogeneity of PAA cryogel in terms of complexation properties toward Cu(II) ions. Even at a low cross-linking degree, very fast sorption centers with the sorption rate constant logarithm (Ks) > −0.5 were not detected for PAA cryogel. In Figure 5a, one can also see centers with virtually irreversible sorption of Cu(II) ions in PEI cryogel (Kd < −4); however, the contribution of such centers to the sorption properties of PAA is rather low, so that the maximal difference in Cu(II) affinity constants of PAA and PEI cryogel was about 2 logarithm units.

Using DFT calculations, we have earlier shown that the relative formation energies (∆E’) for the Cu(II) complexes with PAA and PEI (geometrically optimized structures are shown in Figure 5b) were equal to −175 and −227 kJ/mol confirming the higher complexation power of PEI. This difference in affinities of PEI and PAA cryogels significantly affects the efficiency of Cu(II) uptake from the solutions containing other complexing agents. Figure S2, Supplementary Materials shows that the PAA cryogel was not efficient in recovering Cu(II) ions from 1 M solution of ammonia acetate, while PEI cryogel allowed Cu(II) removal in fixed-bed to the level recommended by the WHO for drinking water (speciation for Cu(II) ions for the sorption conditions in given in Table S2, Supplementary Materials). Although the breakthrough curve on the PEI cryogel was less steeper than that in water, the dynamic sorption capacity until breakthrough point for Cu(II) in 1 M solution of ammonia acetate was 78% of that in water. PEI cryogels were efficient for the recovery of Cd(II), Zn(II), Ni(II), and Co(II) ions [15,31] from single- and multi-component (Figure S3a, Supplementary Materials) solutions, while the PAA cryogel was not efficient for the uptake of Ni(II) and Cd(II) ions (Figure S3b, Supplementary Materials), which can be a disadvantage for the treatment of waste streams containing different heavy metal ions, but an advantage in metal ions separation.

**Figure 6:**
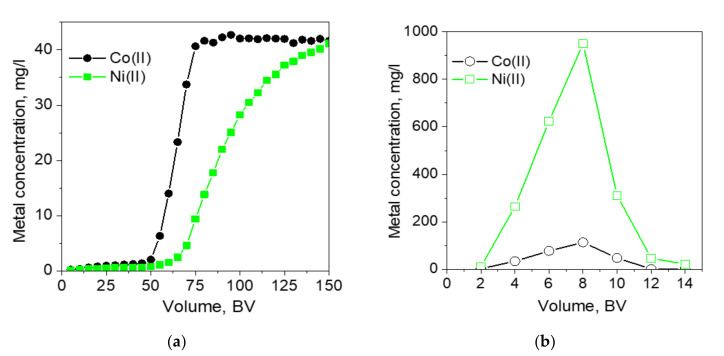
Breakthrough curves of Ni(II) and Co(II) ions sorption on monolith PEI cryogel, initial metal concentrations are 50 mg/L, flow rate is 8 bed volumes (BV)/h, column diameter is 0.48 cm, bed length is 6 cm (**a**). Metal ion elution curve from the PEI cryogel with 0.1 M HNO_3_ solution, flow rate of 16 BV/h (**b**).

Table 1 shows the comparative selectivity of PAA and PEI cryogels in two component solutions containing Cu(II), Zn(II), and Cd(II) ions in a batch under the equilibrium conditions. The high affinity of PEI for many metal ions results in the much lower selectivity of the PEI cryogel compared to that of PAA, which shows high selectivity of Cu(II) sorption even from mixtures containing Zn(II) ions, which can be efficiently adsorbed by PAA from a one-component solution (Figure 4b). PAA has very low sorption capacity toward Co(II) and Ni(II) ions, but both ions can be efficiently recovered from a two-component solution (Figure 6a); however, only Ni(II) ions can be efficiently eluted from the sorbent phase with 0.1 M HNO_3_ solution (Figure 6b). The non-reversible character of Co(II) sorption was earlier reported for a PEI–silica composite [43] and was related to the slow oxidation of Co(II) to Co(III) with dissolved oxygen resulting in the formation of highly stable Co(III)–PEI complexes.

$$K_{s}=\frac{(C_{M1}^{0}-C_{M1}^{e})\cdot C_{M2}^{e}}{C_{M1}^{e}\cdot (C_{M2}^{0}-C_{M2}^{e})}$$

### 2.3. Composite Cryogels for Cesium Ion Sorption

As mentioned in the introduction, the stability of metal ion/polymer ligand complexes was an important parameter for the in situ synthesis of organic–inorganic hybrids. Taking into account the difference in affinities of PAA and PEI cryogels toward Cu(II) ions, we have investigated how a polymer matrix affects the efficiency of composite sorbents containing Cu(II) ferrocyanide for cesium ion removal. Transition metal ferrocyanides are the most efficient materials for selective uptake of cesium radionuclides from liquid radioactive wastes and natural waters; however, due to their small size and low mechanical strength, they cannot be used in columns without immobilization in organic or inorganic matrices, which can be realized under dynamic conditions [44,45,46,47]. Polymeric ligands can be used for the fabrication of such composite sorbents with several routes, in most cases, mechanically stable granulated materials were obtained by co-precipitation of polymer with transition metal ions and potassium ferrocyanide [48,49], but an inorganic phase can be also formed in situ via sequential loading of transition metal ions and [Fe(CN)_6_]^4−^ anion [33,50]. We have demonstrated the feasibility of the latter approach for the fabrication of supermacroporous monolith sorbents on cryogel of carboxyethylchitosan [33], but PAA and PEI cryogels can be cheaper alternatives to the more expensive carboxyalkylchitosan-based matrices. Although chitosan derivatives definitely have advantages for drinking water treatment applications, in composites with inorganic materials targeted to the sorption of hazardous species, the positive impact of non-toxicity and biodegradability of chitosans as matrices becomes much less important. Moreover, the high positive charge of the PAA and PEI cryogel surfaces can be used for electrostatically driven precipitation of the copper ferrocyanide colloids (CuFCN) in cryogel supermacropores. Here, both fabrication methods were used, which are further referred to as fabrication in situ (sequential loading of PAA and PEI cryogels with Cu(II) and ferrocyanide ions) and “precipitation” (loading of PAA cryogel with preformed Cu_2_[Fe(CN)_6_] colloids).

**Figure 7:**
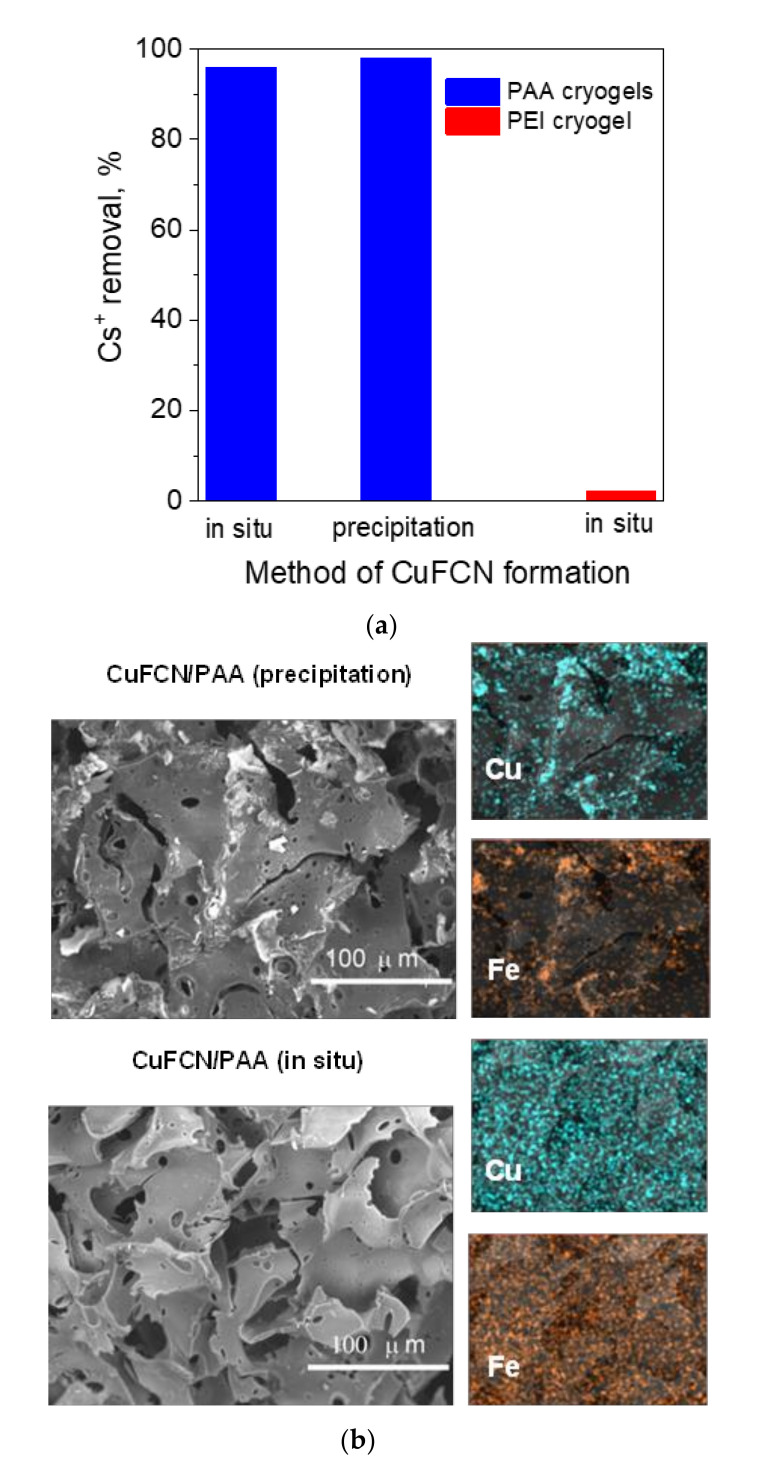

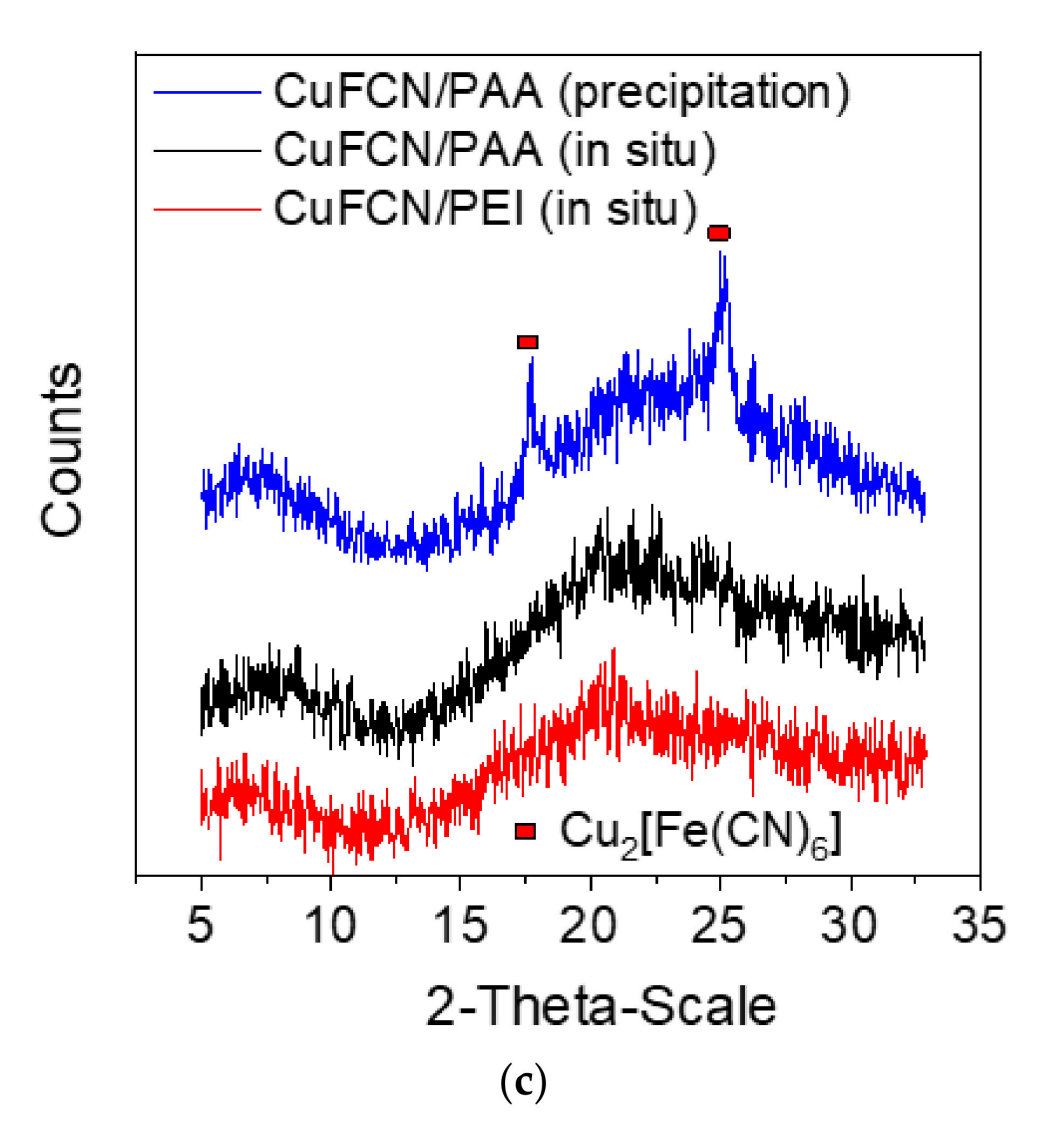
Efficiency of Cs^+^ ion uptake with composite sorbents fabricated by the precipitation of ex situ formed copper ferrocyanides (CuFCN) on PAA cryogel or in situ formation in PAA and PEI cryogels, initial Cs concentration 20 mg/L, solid:liquid ratio of 1:1000 (**a**). SEM images and energy dispersive X-ray (EDX) mapping for CuFCN/PAA composite cryogels fabricated via CuFCN precipitation and in situ formation (**b**). XRD patterns for CuFCN/ PAA and CuFCN/PEI composite cryogels (**c**).

Figure 7a shows that both types of composite PAA cryogels allowed the removal of 96–98% of cesium ions from a solution with cesium concentration of 20 mg/L, while the efficiency of the PEI-based composite fabricated in situ was significantly lower. SEM-EDX analysis (Figure 7b and Figure S4, Supplementary Materials) confirmed a macroporous structure of the composites and a difference in CuFCN distribution depending on the composite fabrication method. Although precipitation of the ex situ formed CuFCN colloids yielded a material with heterogeneous distribution of inorganic phase, as can be seen from energy dispersive X-ray (EDX) mapping of the CuFCN/PAA (precipitation) cryogel surface, the composite was stable under the flow conditions (Figure S5, Supplementary Materials) and showed no release of ferrocyanide during Cs(I) sorption. Release of Cu(II) ions was related to the ion-exchange mechanism of sorption.

A composite formed in situ had homogeneous distribution of Cu and Fe on the surface, but CuFCN crystallites were not detectable with resolution of the SEM either on PEI or on PAA cryogels. XRD patterns of the composite formed in situ (Figure 7c) did not show CuFCN signals, which were detected for CuFCN/PAA (precipitation) cryogel. This suggests the formation of smaller CuFCN nanoparticles at least in the case of PAA cryogel, which was proved as an efficient sorbent for Cs ions. However, the XRD data did not help to explain the sorption inactivity of the CuFCN/PEI (in situ) composite. EDX analysis (Table S3, Supplementary Materials) did not reveal significant deviation of the Cu/Fe atomic ratio for the CuFCN/PEI composite from theoretical value (Cu/Fe = 2), while the Cu/Fe ratio was above 2 for CuFCN/PAA (in situ) cryogel indicating that not all Cu(II) ions in the PAA matrix were involved in CuFCN formation.

**Figure 8:**
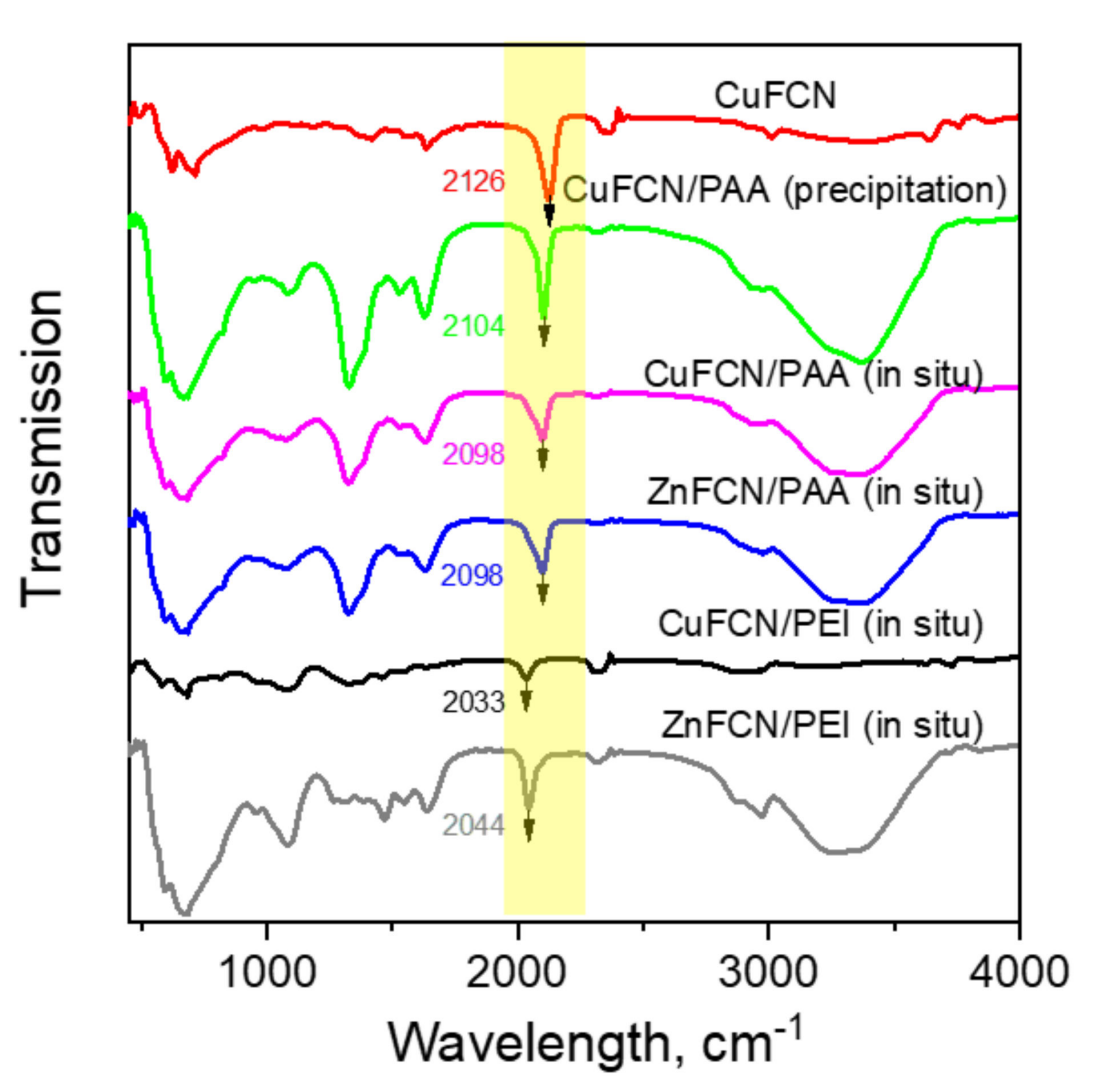
IR spectra of pure Cu(II) ferrocyanide (CuFCN) and composites obtained by precipitation of CuFCN or in situ formation of Cu(II) and Zn(II) ferrocyanide in PAA and PEI cryogels.

Taking into account a difference in the color of CuFCN/PAA (reddish brown) and CuFCN/PEI (dark blue) composites formed in situ, one can suggest that the PEI matrix had a significant effect on formation of Cu(II) ferrocyanide in situ. There are two possible reasons for this: (i) very strong binding of Cu(II) ions by PEI; and (ii) dissociation of [Fe(CN)_6_]^4−^ complex due to the very high Lewis basicity of PEI. The latter possibility was demonstrated earlier for N-containing ligands and Fe(III) cyanide complexes [51]. To elucidate the impacts of these factors, we have recorded IR spectra (Figure 8) of pure copper ferrocyanide (CuFCN) and composites formed in PAA and PEI matrices via precipitation and in situ formation. IR spectra of CuFCN and all composites showed bands at ~590 cm^−1^ typical for Fe(II) ferrocyanide (Fe-CN deformation mode) [52]. The stability of the [Fe(CN)_6_]^4-^ complex ion in the CuFCN/PEI composite was also confirmed by the singlet in the Mössbauer spectrum (Figure S6, Table S4, Supplementary Materials) of CuFCN/PEI (in situ) corresponding to the low-spin Fe(II) in hexacyanoferrates [53].

The most important information on the matrix’s effect was obtained from IR spectra thanks to the high sensitivity of the band at 2040–2100 cm^−1^ (–C≡N stretching vibration) to the type of metal ion in ferrocyanide and composite fabrication method. Figure 8 shows that frequencies of the –C≡N band in CuFCN/PAA were close to the values found for pure CuFCN, with a minor difference between composites formed in situ and by precipitation. In contrast, frequency of –C≡N in CuFCN/PEI (in situ) composite was 93 cm^−1^ lower than in CuFCN. Since relatively high frequency for the –C≡N band in the copper ferrocyanide was attributed to a particularly strong interaction between the copper atom and the –C≡N ligands due to the ability of Cu^2+^ to receive electrons from -CN group to adopt the 3d^10^ configuration [53]. Cu(II) complexation by PEI as a strong N-donor ligand can, probably, result in formation of a cyanide-bridged complex [54] rather than a crystal phase of copper ferrocyanide. This can explain a poor sorption activity of the CuFCN/PEI (in situ) composite toward cesium, despite the ideal stoichiometric atomic ratio Cu/Fe = 2, which can be expected for CuFCN. Replacement of Cu(II) in the polymer matrix with Zn(II) ion, which forms less stable complexes with polyamines, had no effect on CN band position in PAA-based composite but resulted in its shift to the higher frequency in the PEI-matrix (Figure 8). It should be mentioned that –C≡N frequency in ZnFCN/PEI was still lower than that observed usually in Zn(II) ferrocyanides [52,53] that can be related to the changes in Zn(II) coordination from tetrahedral (typical for Zn(II) ferrocyanides) to octahedral [53,55]. Thus, we can conclude that strong binding of transition metal ions by PEI as multidentate N-donor ligand has a negative effect on the formation of the crystalline phase of transition metal ferrocyanides capable of selectively adsorbing cesium ions.

## 3. Materials and Methods

### 3.1. Materials

Branched polyethyleneimine (PEI) of an average molecular weight of 25 kDa and poly(allylamine hydrochloride) (PAA) with molecular weight from 120 to 200 kDa was purchased from “Alfa Aesar”. 1,4-butanediol diglycidyl ether (DGEBD) was purchased from Sigma–Aldrich (Darmstadt, Germany). Other reagents were of analytical grade.

### 3.2. PAA and PEI Cryogel Fabrication

PEI was cross-linked in 5% solution (pH 10) with DGEBD at a molar ratio to PEI (monomer unit) of 1:4 as described in detail in [15]. PAA was cross-linked in 5% solution (pH 7.4) with DGEBD at molar ratios to PAA (monomer unit) from 1:2 to 1:12 using the same procedure as described for PEI. To fabricate monolith PAA and PEI cryogels, polymer solutions with a cross-linker agent were placed into insulin syringes of an inner diameter of 4.8 mm and kept frozen for 7 day at −20 °C. After thawing, the monolith cryogels were washed with 10 mL of 0.1 M NaOH solution and distilled water (until neutral pH) using a peristaltic pump (Ismatec, Wertheim, Germany) and sealed in the swollen state until used. The plate-like fines of a thickness of ~10 µm and linear dimensions of ~100 µm were obtained by ultrasound treatment of the swollen PAA cryogel to investigate Cu(II) ion sorption kinetics.

Swelling of the cross-linked PAA cryogels was determined from the difference in weights of the cryogels that were swollen for 24 h in distilled water and the dry fragments of the monolith cryogels. To determine the contribution of weakly bound water (predominantly from macropores) to the swelling, the swollen cryogels were first finger-squeezed and weighed. The maximal flow rate (mL/h) was determined by passing distilled water through the columns with monolith cryogels (inner diameter 4.8 mm, bed height of 6 cm), then the flow rate was recalculated to bed volumes per hour (BV/h).

### 3.3. Morphology of Cryogels

The structure of the swollen (never dried) PAA cryogels was investigated using a Carl Zeiss LSM 780 confocal laser scanning microscope (Oberkochen, Germany) with a regular 20× objective lens. Before the measurements, thin cuts of cryogels were stained with fluoresceine in 0.075 mM aqueous solution, the contact time was 72 h, then the material was thoroughly washed with distilled water. The excitation and emission wavelengths used were set at 488 and 530 nm, respectively. The average pore size and wall thickness were calculated using the ImageJ software [56].

### 3.4. Rheological Properties

The mechanical spectra of PAA and PEI 5% solutions were recorded after the addition of cross-linker at a DGEBD:polymer molar ratio of 1:4 under constant stirring and keeping solutions for 24 h at +25 or −20 °C. The spectra were recorded in the frequency range between 1 and 100 Hz at a temperature of 25 °C and constant strain 2% using a Physica MCR 301 rheometer (Anton Paar GmbH, Graz, Austria) with a plate–plate measuring system of a diameter of 25 mm, the storage and loss moduli as well as complex viscosity were calculated from the frequency sweeps (mechanical spectra) using the equipment software.

### 3.5. Investigations of the Sorption Properties of PEI and PAA Cryogel

The kinetics of Cu(II) sorption on fines of PAA cryogel was studied at a solid:liquid ratio of 1:1000 from aqueous solutions of Cu(NO_3_)_2_ of an initial concentration of Cu(II) −76.5, 128, and 255 mg/L, pH = 5. The cryogel fines were placed into the permeable for solution closed bags, which were inert toward Cu(II) ions sorption, and constantly agitated using a Biosan PSU-20i orbital shaker (Riga, Latvia) at 290 rpm, the sampling of the solution was performed regularly over 3000 min. The copper concentration in the solutions was determined by atomic absorption spectrometry (AAS) using a Solaar M6 (Thermo Scientific, Waltham, MA, USA) device. The same procedure was used for the investigation of Cu(II) ion sorption on PEI cryogels. Maximal sorption capacities were determined from the Cu(II) ion sorption isotherms (not shown) obtained in aqueous Cu(NO_3_)_2_ solutions, pH 5, at the solid:liquid ratio of 1:1000, the contact time was 3000 min, the solutions with sorbents were agitated under the same conditions as in the kinetics study. The adsorbed amounts were calculated using the difference in initial and equilibrium concentrations of the metal ions in the solutions as determined by AAS.

The dynamics of Cu(II) and Zn(II) ion sorption on monolith PAA and PEI cryogels were investigated by feeding an aqueous solution of Cu(NO_3_)_2_ containing 100 mg/L of Cu(II) or Zn(NO_3_)_2_ with Zn(II) concentration of 75 mg/L through a syringe with 1 mL of the swollen cryogel (inner diameter—4.8 mm, bed length—6 cm) at a flow rate of 84 or 168 BV/h. Samples were collected every 5 mL; copper concentrations were determined with AAS. The metal ion sorption from multicomponent systems was studied using the same protocol; initial concentrations and conditions are given in the figure captions.

Contribution of physical sorption to the overall sorption capacity was investigated on PEI and PAA cryogels saturated with Cu(II) under dynamic conditions. The monolith cryogels were washed with distilled water in columns, removed, and air-dried. Five milligrams of the sorbents with known copper content was immersed into 5 mL of distilled water and left for 96 h under constant agitation, then Cu(II) concentration in the solution was determined with AAS. Physical sorption was calculated as % of copper released from the sorbent phase to the solution.

### 3.6. Fabrication of the Composite Cryogels Containing Copper Ferrocyanide (CuFCN) and Investigations of Their Sorption Properties

Composite cryogels containing CuFCN were fabricated by (i) precipitation of ex situ formed CuFCN colloids on PAA-DGEBD 1:8 cryogel, and (ii) in situ formation of CuFCN in PAA-DGEBD 1:6 and PEI-DGEBD 1:4 cryogels. The composite formed by precipitation method was obtained as follows: 0.5 mL of 0.1 M Cu(NO_3_)_2_ solution was added to 50 mL of distilled water, then 0.25 mL of 0.1 M K_4_[Fe(CN)_6_] solution was added dropwise under constant stirring, the obtained colloid solution of reddish brown color was fed through the column with 1 mL of PAA cryogel at a flow rate of 122 BV/h using a peristaltic pump (the stirring was continued). The CuFCN colloids were distributed over the full length of the column; iron and copper in outlet solutions were not detected by AAS. The column with the composite material (CuFCN/PAA (precipitation) cryogel) was washed with distilled water and used to investigate the dynamics of Cs^+^ ion sorption and Cs^+^ uptake in a batch. In situ formation of composites containing CuFCN was performed as follows: PAA and PEI cryogels were saturated with Cu(II) ions under dynamic conditions and washed with distilled water; in a week, 10 mL of 0.01 M K_4_[Fe(CN)_6_] solution was fed through the column at a flow rate of 8 BV/h. Finally, the composites were washed with 150 mL of distilled water, removed from the column, and air-dried for investigation of Cs+ uptake in a batch.

The efficiency of Cs^+^ ion uptake in a batch was determined as follows: 5 mg of the composite cryogel was shaken for 24 h with 5 mL of CsCl solution containing 20 mgCs/L (pH~6). The equilibrium content of cesium was determined by AAS. Dynamics of Cs^+^ ions sorption on CuFCN/PAA (precipitation) cryogel was studied by feeding CsCl solution containing 20 mgCs/L (pH~6) through 1 mL of monolith in insulin syringe at the flow rate of 84 BV/h using a peristaltic pump, the filtrate was collected every 5 mL and analyzed for cesium, copper, and iron content by AAS.

Morphology investigations and elemental analysis of the freeze-dried composite cryogels were performed using a Hitachi TM3000 scanning electron microscope (SEM) equipped with a Bruker Quantax 70 energy dispersive X-ray (EDX) spectrometer at an accelerating voltage of 15 kV.

X-ray powder diffraction analysis (XRD) of composite cryogels was carried out on a Dron-3 multipurpose diffractometer (JSC “Bourevestnik”, St. Petersburg, Russia).

## 4. Conclusions

Here we have investigated the reactivity of affordable and commercially available polyamine PAA (polyallylamine) in cross-linking reaction with diglycidyl ether of 1,4-butandiol and showed that PAA cryogels were formed at lower molar ratios of cross-linker to polymer and had better stability under the flow conditions than did PEI cryogels. Both PEI and PAA cryogels were efficient for the removal of Cu(II) and Zn(II) ions in batch and fixed bed applications to the level set by the WHO for drinking water.

Although PAA cryogels had worse chelating properties than PEI cryogels, they provided high selectivity of Cu(II) recovery over Zn(II) and Cd(II) ions, and Zn(II) ions over Cd(II) ions under equilibrium sorption conditions. PEI cryogels can be used for Co(II) and Ni(II) recovery from two component solutions in fixed-bed and selective elution of Ni(II) ions with 0.1 M HNO_3_ solution.

We have also demonstrated that formation of stable Cu(II)–PEI complex hinders in situ formation of Cu(II) ferrocyanide that results in very poor sorption efficiency of this PEI-based composite in cesium ion uptake. In contrast, PAA cryogels are applicable for composite formation via loading of ex situ formed Cu(II) ferrocyanide colloids or the in situ formation of nanosorbent with preservation of the supermacroporous structure of the matrix.

## Figures and Tables

**Table 1:** Selectivity of PAA and PEI cryogels in two-component mixtures of metal ions (M1 and M2).

Sorbent			C^0^, mg/L	C^e^, mg/L	Sorption, mmol/g	Ks *(M1/M2)
PAA cryogel DGEBD: PAA 1:10	M1	Zn(II)	126	36	1.30	57
	M2	Cd(II)	214	205	0.076	
	M1	Cu(II)	130	15	1.79	236
	M2	Zn(II)	130	126	0.062	
	M1	Cu(II)	131	16	1.66	551
	M2	Cd(II)	233	230	0.025	
PEI cryogel DGEBD:PEI 1:4	M1	Zn(II)	135.8	89	0.71	0.81
	M2	Cd(II)	231	141	0.79	
	M1	Cu(II)	117	6.3	1.66	64
	M2	Zn(II)	137.7	116	0.32	
	M1	Cu(II)	120.6	7.9	1.76	130
	M2	Cd(II)	231.9	209	0.20	

* Ks—the selectivity coefficient was calculated as follows (C^0^ and C^e^ were expressed in mg/L):

## Supplementary Materials

The following are available online, Figure S1: Kinetic curves of Cu(II) ions sorption on fines of PEI cryogel cross-linked with DGEBD at molar ratio DGEEB:PEI 1:4, Ph = 5, T = 25 °C, initial Cu(II) concentration 0.78 mmol/L. Figure S2: Breakthrough curves of Cu(II) ions sorption on PEI and PAA monolith cryogels from water and 1 M CH_3_COONH_4_ solution, initial Cu(II) concentration (C_0_) is 100 mg/L, pH = 5, flow rate is 84 BV/h, monolith volume is 1 mL. Figure S3: Breakthrough curves of Zn(II), Cd(II), and Cu(II) ions sorption on PEI-DGEBD 1:4 cryogel from a three-component mixture in water, pH 5, initial metal concentrations were 51, 39, and 30 mg/L, respectively (a). Breakthrough curves of Zn(II), Cd(II), Cu(II), and Ni(II) ions on PAA-DGEBD 1:8 cryogel from four-component mixture in water, pH 5, initial metal concentrations were 35, 34, 29, and 32 mg/L, respectively (b). Column parameters for (a) and (b): diameter is 0.48 cm, bed length is 6 cm, flow rate is 84 BV/h. Figure S4: SEM images and EDX mapping for CuFCN/PEI composite cryogel fabricated in situ. Figure S5: Breakthrough curve of Cs^+^ ion sorption on CuFCN/PAA (precipitation) cryogel from CsCl solution containing 20 mgCs/L (pH~6) and release of Cu(II) and Fe(III) species to outlet solution, monolith composite volume is 1 mL, flow rate is 84 BV/h. Figure S6: Mössbauer spectra of CuFCN/PEI (in situ), CuFCN, K_4_Fe(CN)_6_ samples: dots are experimental data, lines are spectra fits obtained with the WinNormos program. Table S1: Cross-linking conditions for PAA and PEI cryogel fabrication and cryogel characteristics. Table S2: Speciation of Cu(II) ionic forms in 1 M CH_3_COONH_4_ solution and water. Calculations were performed using chemical equilibrium model Visual MINTEQ ver.3.0. Table S3: Elemental analysis of CuFCN-containing cryogels (SEM-EDX data). Table S4. Mössbauer parameters for Cu(II) ferrocyanide and composite, T = 298 K.
