# Supplementary material for: Rational Design of Polyamine-Based Cryogels for Metal Ion Sorption
Source: Molecules. 2020 Oct 19;25(20):4801. doi: 10.3390/molecules25204801 (PMC7587524; doi:10.3390/molecules25204801)
Supplement: Supplementary file 1 [file molecules-25-04801-s001.pdf]

Supplementary materials

Rational Design of Polyamine-Based Cryogels for Metal Ions Sorption

Irina Malakhova[1], Yuliya Privar[1], Yuliya Parotkina[1], Aleksandr Mironenko[1], Marina Eliseikina[2], Denis Balatskiy[1], Alexey Golikov[1], and Svetlana Bratskaya[1]*

[1] Institute of Chemistry, Far Eastern Branch of Russian Academy of Sciences, 159, prosp.100-letiya Vladivostoka, Vladivostok 690022, Russia; sbratska@ich.dvo.ru

[2] A.V. Zhirmunsky National Scientific Center of Marine Biology, Far Eastern Branch of Russian Academy of Sciences, 17, Palchevskogo street, Vladivostok 690041, Russia

* Correspondence: sbratska@ich.dvo.ru

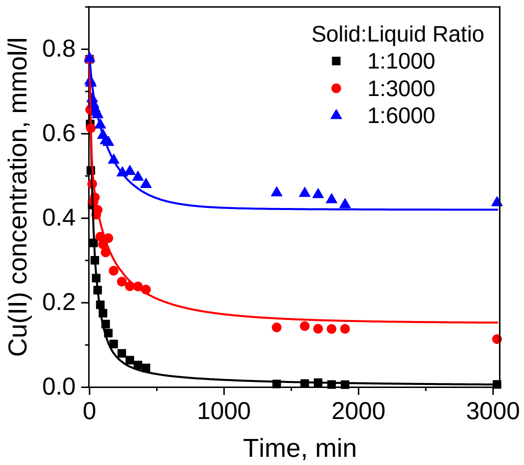

Figure 1S. Kinetic curves of Cu(II) ions sorption on fines of PEI cryogel cross-linked with DGEBD at molar ratio DGEEB:PEI 1:4, pH=5, T=25°C, initial Cu(II) concentration 0.78 mmol/l.

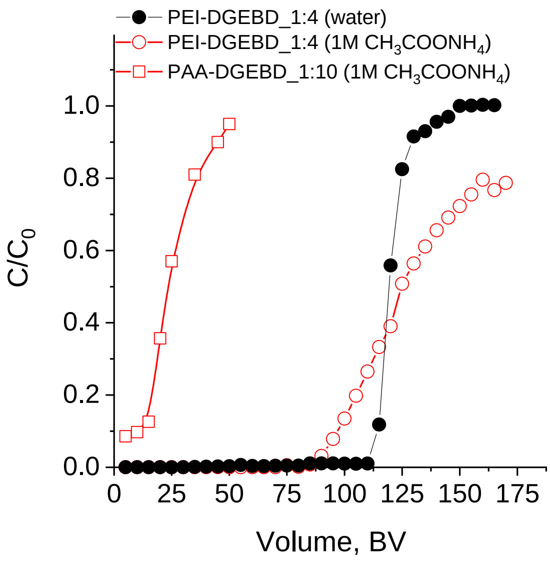

Figure 2S. Breakthrough curves of Cu(II) ions sorption on PEI and PAA monolith cryogels from water and 1M CH_3COONH_4 solution, initial Cu(II) concentration (C_0) is 100 mg/l, pH=5, flow rate is 84 BV/h, monolith volume is 1 ml

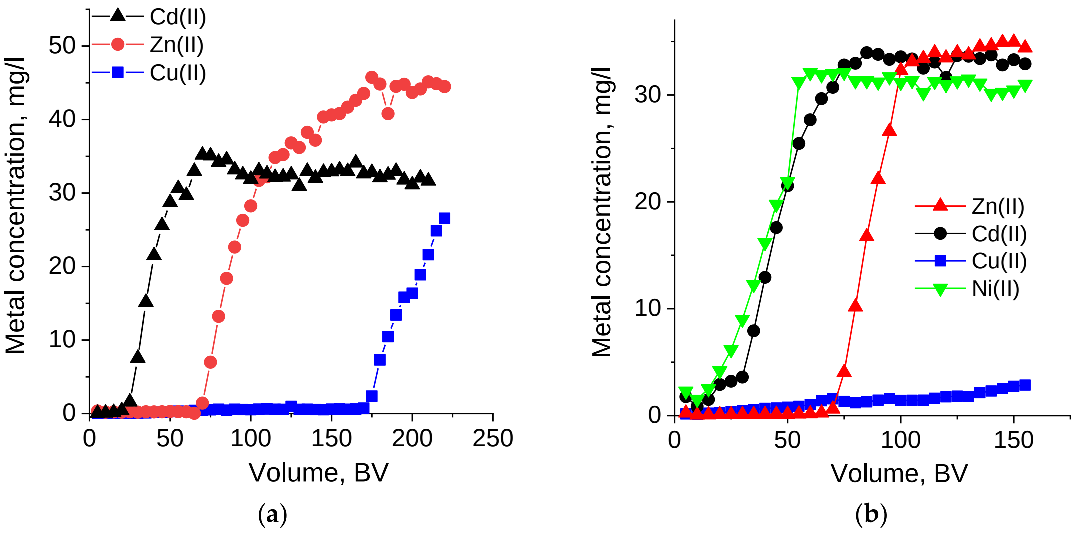

Figure 3S. Breakthrough curves of Zn(II), Cd(II), and Cu(II) ions sorption on PEI-DGEBD 1:4 cryogel from three-component mixture in water, pH 5, initial metal concentrations were 51 mg/l, 39 mg/l and 30 mg/l, respectively (a). Breakthrough curves of Zn(II), Cd(II), Cu(II), and Ni(II) ions on PAA-DGEBD 1:8 cryogel from four-component mixture in water, pH 5, initial metal concentrations were 35 mg/l, 34 mg/l, 29mg/l, and 32 mg/l, respectively (b). Column parameters for (a) and (b): diameter is 0.48 cm, bed length is 6 cm, flow rate is 84 BV/h.

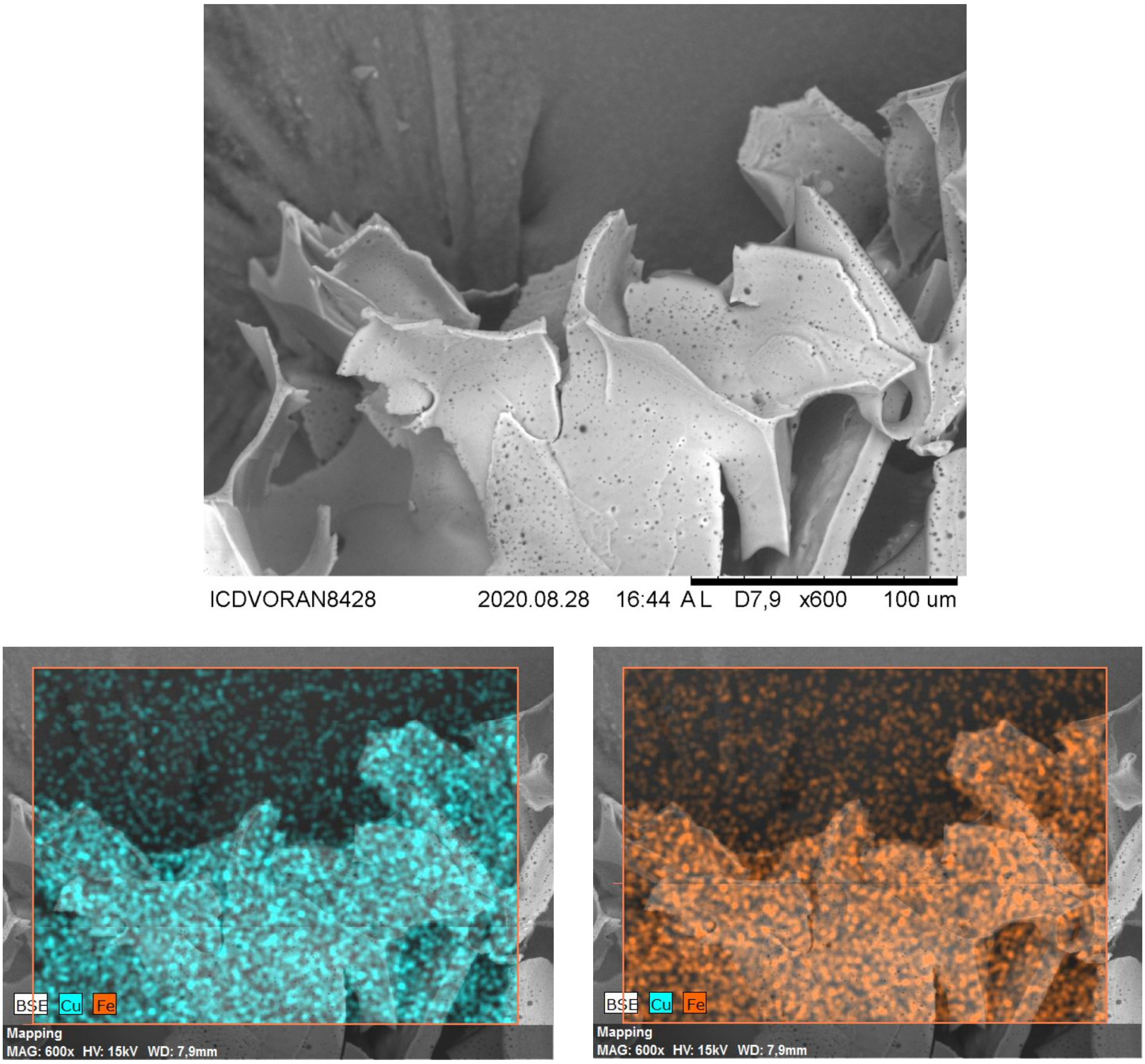

Figure 4S. SEM images and EDX mapping for CuFCN/PEI composite cryogel fabricated in situ

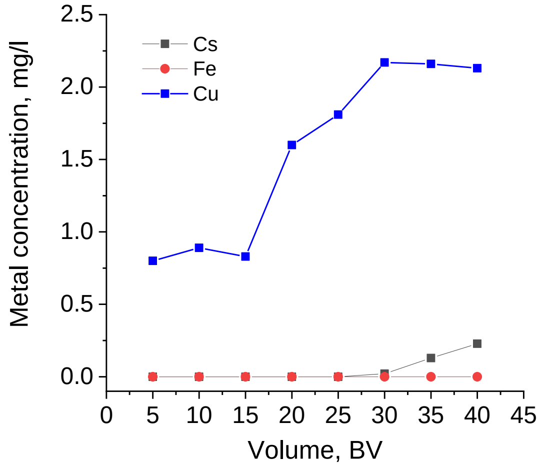

Figure 5S. Breakthrough curve of Cs^+ ions sorption on CuFCN/PAA (precipitation) cryogel from CsCl solution containing 20 mgCs/l (pH~6) and release of Cu(II) and Fe(III) species to outlet solution, monolith composite volume is 1 ml, flow rate is 84 BV/h.

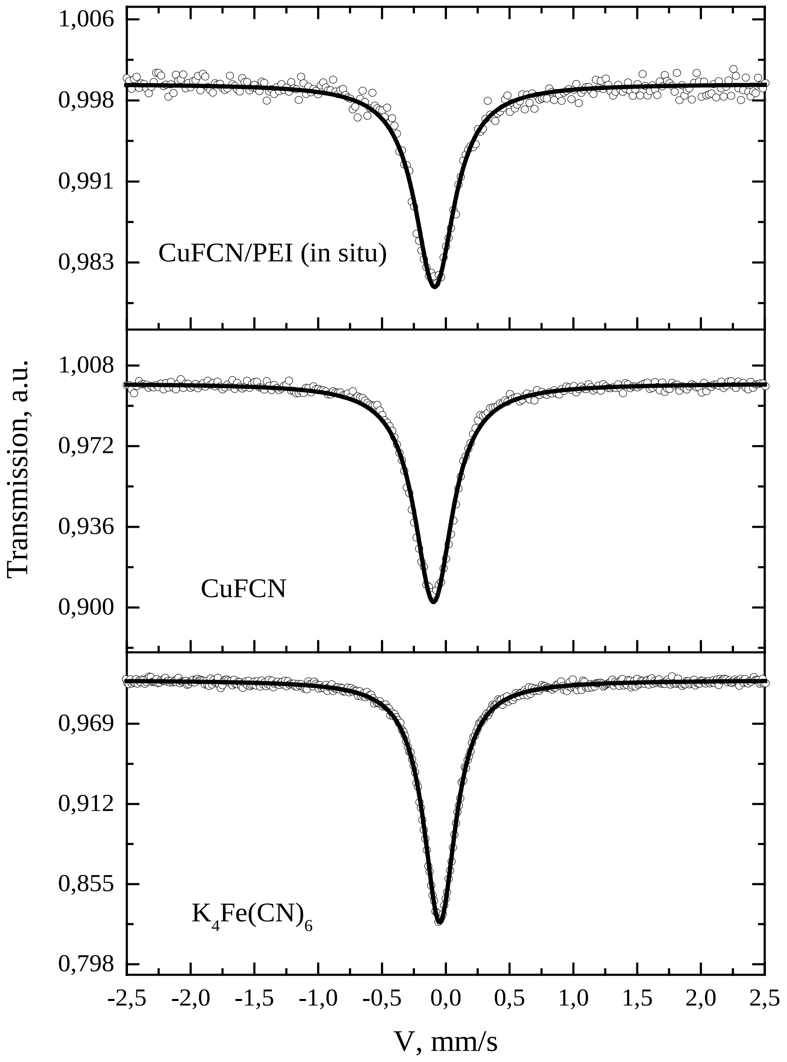

Figure 6S. Mössbauer spectra of CuFCN/PEI (in situ), CuFCN, $K_4Fe(CN)_6$ samples: dots are experimental data, lines are spectra fits obtained with the WinNormos program

The Mössbauer spectra were obtained at room temperature using a Wissel spectrometer in transmission geometry and a $^{57}Co(Rh)$ source. The Mössbauer spectra were fitted using the WinNormos program in order to obtain the values of isomer shift (δ), quadrupole splitting (Δ), linewidth (Γ) and relative sub-spectrum area (see Table 4S). The velocity scale was calibrated using the spectrum of metallic iron (α-Fe). The value of isomer shifts was determined relative to the center of gravity of the α-Fe spectrum.

Table 1S. Cross-linking conditions for PAA and PEI cryogels fabrication and cryogels characteristics

Molar ratio DGEBD:polymer	Weight of 5% polymer solution, g	Weight of DGEBD, g	Maximal flow rate, BV/h	Static sorption capacity for Cu(II) ions, mg/g
		**PEI**		
1:1	10	1.3271	377	105
1:2	10	0.6635	443	121
1:4	10	0.3317	485	172
1:6	10	0.2211	9	221
		**PAA**		
1:2	10	1.4784	307	63
1:4	10	0.7392	485	175
1:6	10	0.4928	485	162
1:8	10	0.3696	485	190
1:10	10	0.2956	485	198
1:12	10	0.2461	485	214

Table 2S. Speciation of Cu(II) ionic forms in 1M CH_3COONH_4 solution and water. Calculations were performed using chemical equilibrium model Visual MINTEQ ver.3.0.

**1M CH3COONH4, pH=5, Cu 100 mg/L (0.00156 M)**		**Water, pH=5, Cu 100 mg/L (0.00156 M)**	
Cu(II) forms	%	Cu(II) forms	%
Cu^{+2}	0.101	Cu^{+2}	98.806
$[Cu(Acetate)_2]$ (aq)	23.328	$[Cu(NO_3)]^+$	0.371
$[Cu(Acetate)_3]^-$	72.282	$[Cu(OH)]^+$	0.513
$[Cu(NH_3)]^{+2}$	0.047	$[Cu_2(OH)]^{+3}$	0.013
$[Cu(Acetate)]^+$	4.236	$[Cu_2(OH)_2]^{+2}$	0.297

Table 3S. Elemental analysis of CuFCN-containing cryogels (SEM-EDX data)

Polymer matric (method of preparation)	Cu, at%	Fe, at %	Cu/Fe Atomic Ratio	Average Cu/Fe atomic ratio
**PAA-DGEBD 1:6 cryogel**	71.98	28.02	2.57	2.5±0.2
**(in situ)**	72.18	27.82	2.59	
	69.10	30.90	2.23	
**PEI-DGEBD 1:4 cryogel**	66.37	33.63	1.97	2.0±0.3
**(in situ)**	60.80	39.20	1.55	
	65.34	34.66	1.88	
	69.41	30.59	2.27	
	64.10	35.90	1.78	
	71.26	28.74	2.48	
	66.31	33.69	1.974	
	65.58	34.42	1.90	
	69.35	30.65	2.26	
**PAA-DGEBD 1:8 cryogel**	65.10	34.90	1.86533	2.0±0.3
**(precipitation)**	66.56	33.44	1.99043	
	70.28	29.72	2.36474	

Table4S. Mossbauer parameters for Cu(II) ferrocyanide and composite, T=298K*

Compound	*δ* (mm/s)	*Δ* (mm/s)	*Γ* (mm/s)	Assignment
CuFCN/PEI (in situ)	-0.09	–	0.38	Low spin Fe(II)
CuFCN	-0.10	–	0.36	Low spin Fe(II)
$K_4Fe(CN)_6$	-0.05	–	0.31	Low spin Fe(II)

[a] isomer shift (*δ*), quadrupole splitting (*Δ*), linewidth (*Γ*). Values of *δ* are reported relative to α-Fe metal. Fitting error in the values of *δ*, *Δ* and *Γ* remained below 0.01 mm/s.
